# Efficacy and safety of PD-1/ PD-L1 inhibitors as adjuvants in the treatment of patients with solid cancers: A systematic review and meta-analysis of randomized controlled trials

**DOI:** 10.18632/oncotarget.28855

**Published:** 2026-03-31

**Authors:** Maryam Aleid, Fatimah Aleid, Daniah Allbdi, Ahmad Rchdeih, Dhai Almuteri, Abdulelah Almesned, Samaa Alotab, Yumna AlMishary, Galia Alsamman, Atlal Abusanad

**Affiliations:** ^1^College of Medicine, Imam Abdulrahman Bin Faisal University, Dammam 34212, Eastern Province, Saudi Arabia; ^2^Faculty of Medicine, King Abdulaziz University, Jeddah 21589, Saudi Arabia; ^3^Internal Medicine Resident, Royal Commission Hospital, Al-Jubail 31961, Saudi Arabia; ^4^Internal Medicine Resident, King Fahad Specialist Hospital, Qassim Health Cluster, Buraydah 52366, Saudi Arabia; ^5^College of Medicine, Qassim University, Unaizah 51911, Saudi Arabia; ^6^College of Medicine, Alfaisal University, Riyadh 11533, Saudi Arabia; ^7^College of Medicine, Majmaa University, Riyadh 15341, Saudi Arabia; ^8^Obstetric and Gynecology Resident, Dr. Suliman Al Habib Women Health, Riyadh 12344, Saudi Arabia

**Keywords:** PD-1, PD-L1, adjuvant immunotherapy, cancer, solid tumor

## Abstract

Background/Objectives: Programmed cell death protein-1 (PD-1) and programmed death ligand-1 (PD-L1) inhibitors are widely used in cancer treatment. Their benefit as adjuvant therapy in solid tumors is still being defined. This systematic review and meta-analysis evaluated the efficacy and safety of PD-1 and PD-L1 inhibitors as adjuvant treatment in patients with solid tumors.

Methods: We conducted a systematic review and meta-analysis of randomized controlled trials in accordance with PRISMA recommendations and PROSPERO registration CRD42024563699. PubMed, Web of Science, Cochrane Library, and Google Scholar were searched for randomized controlled trials published in English that evaluated adjuvant PD-1 or PD-L1 inhibitors in solid cancers.

Results: Thirteen randomized controlled trials published between 2021 and 2023 were included. Adjuvant PD-1 and PD-L1 inhibitors improved disease-free survival (hazard ratio 0.75; 95% CI 0.65–0.86) and distant metastasis-free survival (hazard ratio 0.69; 95% CI 0.54–0.87). No clear difference in overall survival was observed. Trial-level subgroup sizes varied across cancer types.

Conclusions: Adjuvant PD-1 and PD-L1 inhibitors improve disease-free and distant metastasis-free survival in selected patients with high-risk solid tumors. The clinical benefit must be balanced against higher toxicity rates. Because the number of studies within each cancer type remains limited, the strength of cancer-specific conclusions is restricted, and further research is required.

## INTRODUCTION

Cancer accounted for almost 10 million deaths globally in 2020, making it the second leading cause of mortality [1, 2]. Solid tumors account for about 90% of these cancer cases [2]. Among the most prevalent cancers worldwide are breast, lung, colon, rectal, and prostate cancers [3].

Recently, there has been a growing focus on utilizing immunotherapy in the initial phases of specific cancers, driven by the hypothesis that earlier treatment leads to better outcomes [4].

Advances in immunotherapy, including immune checkpoint blockade (ICB), chimeric antigen receptor T-cell therapy, and cancer vaccines, have markedly improved cancer treatment by harnessing the immune system [5]. When applied as adjuvant therapies following primary treatment, these approaches can help eradicate micrometastases, thereby improving cure rates and extending overall survival (OS) [4, 6].

Immune checkpoint inhibitors (ICIs) allow the host immune system to mount an immunological defense against the tumor by reversing the immunosuppressive characteristics of cancer cells. Programmed cell death ligand-1 (PD-L1) and programmed cell death protein-1 (PD-1) represent one of the main pathways targeted by immune checkpoint blockade (ICB). This pathway is triggered by the interaction of PD-1 with its ligand, which is overexpressed in many tumor types. This interaction inhibits T-cell proliferation, leading to cell apoptosis and exhaustion with subsequent immune escape by cancer cells [7].

Monoclonal antibodies targeting PD-1/PD-L1 are now the standard of care for several solid malignancies [7]. Key agents in this class include Nivolumab and Pembrolizumab, which target PD-1, and Durvalumab, Avelumab, and Atezolizumab, which target PD-L1 [5]. Although these drugs have received approval, the safety and efficacy of PD-1/PD-L1 inhibitors as adjuvant therapies require further investigation. This includes assessing their performance across different solid tumor types at early stages, which remains an active area of research.

This systematic review and meta-analysis summarize current randomized evidence on adjuvant PD-1 and PD-L1 inhibitors in solid tumors. We examined disease-free survival, overall survival, distant metastasis-free survival, and adverse events.

## RESULTS

### Eligible studies and quality

**Figure 1:**
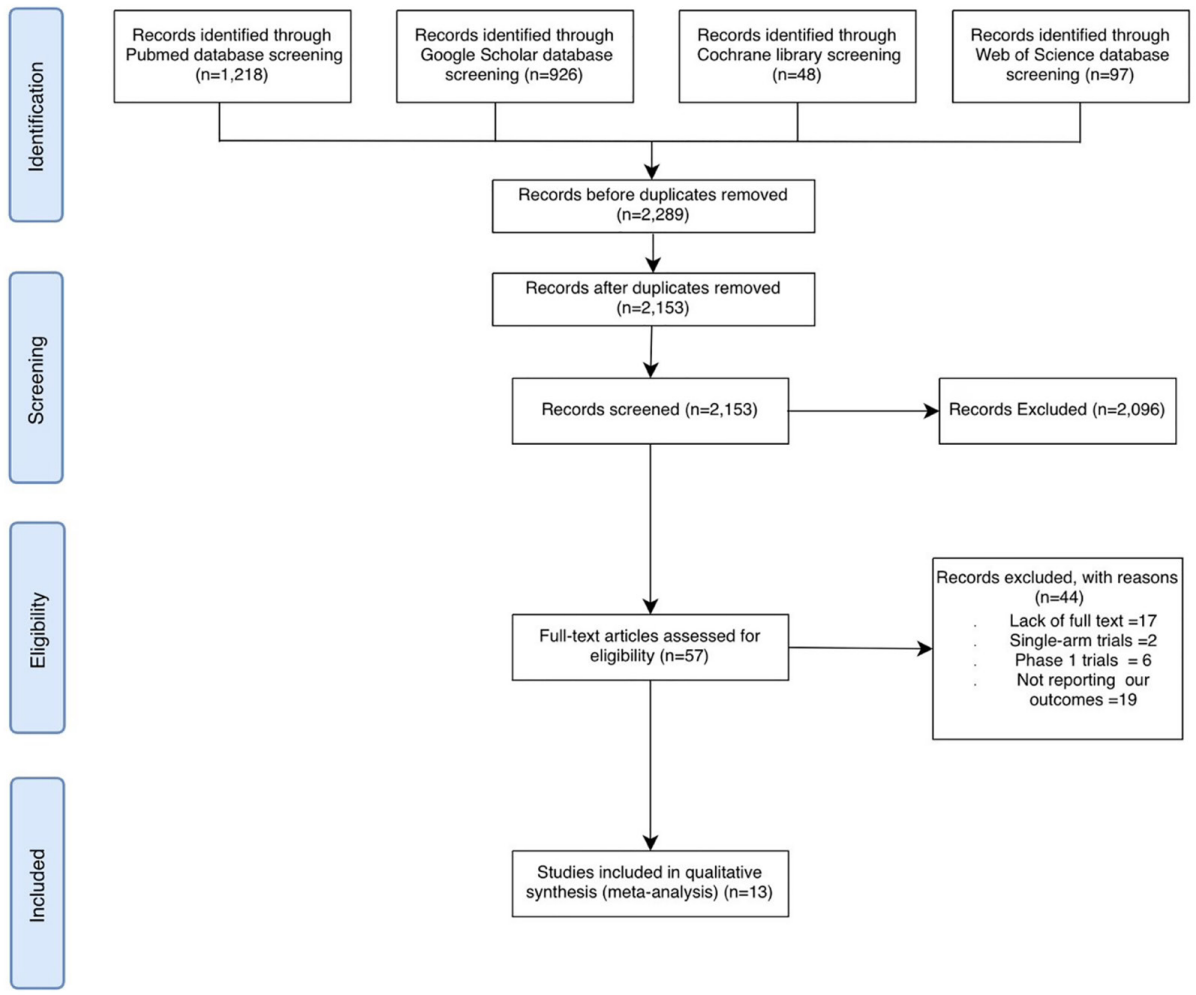
PRISMA flowchart. PRISMA, Preferred reporting items for systematic reviews and meta-analyses.

During the title and abstract screening phase, 2,096 articles were excluded because they did not meet the inclusion criteria. Specifically, these studies did not involve early-stage solid cancers, did not assess PD-1/PD-L1 inhibitors as adjuvant therapy, were not published in English, or were not randomized controlled trials. In addition, 136 duplicate records were removed. The remaining 57 articles underwent full-text evaluation, after which 13 studies were deemed eligible for inclusion (Figure 1).

### Study qualities

A total of 13 randomized controlled trials (RCTs) were included [8–20]. All of the 13 included articles were published in the years of 2021 and 2023. The articles involved participants from Asian (*n* = 9), North America (*n* = 9), South America (*n* = 7) and European regions (*n* = 9). A cumulative sample of 9,850 patients was included. The median age amongst the patients was similar and ranged from 53 to 67 years. The majority of 11 studies were phase III trials and 2 studies conducted phase II trials. The studies investigated the treatment of Renal Cell Carcinoma (*n* = 4), melanoma (*n* = 3), non-small cell carcinoma (*n* = 3), esophageal or gastroesophageal cancer (*n* = 2), and urothelial carcinoma (*n* = 1). The studies involved different PD-1 inhibitors including the PD-1 inhibitor Atezolizumab (*n* = 4), Pembrolizumab (*n* = 4), Durvalumab (*n* = 1), Nivolumab (*n* = 3), and Toripalimab (*n* = 1). (Table 1) demonstrates the characteristics of the included studies.

**Table 1:** Characteristics of the included studies

Study ID	Country	Study phase (II, III)	Cancer type	Cancer stage	Sample size	Age (years)		Gender (Males)		Follow up duration (month)	PD-1 inhibitor used
						PD-1 inhibitor	Control	PD-1 inhibitor	Control		
**Kelly 2021**	International	III	esophageal or gastroesophageal junction cancer	II and III	794	62 (26–82)	61 (26–86)	449 (84)	222 (85)	24.4	Nivolumab
**Bellmunt 2021**	International	III	Urothelial carcinoma	III and IV	809	67 (60–72)	66 (60–73)	322 (79%)	316 (78%)	21.9	Atezolizumab
**Grossman 2022**	United States, Canada, and Ireland	III	Melanoma	III and IV	1,301	53 (20, 82)	54 (18, 86)	383 (59)	395 (60)	47.3	Pembrolizumab
**Motzer 2023**	International	III	Renal cell carcinoma	Variable	816	58 (51, 65)	57 (50, 65)	286 (71)	294 (72)	37	Nivolumab
**Kenmotsu/J 2022**	Japan	III	Non-small cell lung carcinoma	I–III	117	64 (40–75)	68 (37–74)	31 (75.6)	27 (81.8)	38.2–38.3	Atezolizumab
**E.Felip 2021**	International	III	Non-small cell lung carcinoma	IB–IIIA	1005	62 (57–67)	62 (56–68)	337 (66.5%)	335 (67.1)	32.2	Atezolizumab
**E.Felip 2023**	International	III	Non-small cell lung carcinoma	II–IIIA	1005	62 (55–67)	62 (56–67)	89 (77.4)	78 (68.4)	45.3	Atezolizumab
**Choueiri 2021**	International	III	Renal cell carcinoma	II and III	994	60	60	347 (69.9)	359 (72.1)	24	Pembrolizumab
**Choueiri/PRO 2023**	International	III	Renal cell carcinoma	Variable	994	60.0 (27−81)	60.0 (25−84)			11.1	Pembrolizumab
**Powles 2022**	International	III	Renal cell carcinoma	I	994	60 (51–66)	60 (52–67)	347 (70%)	359 (72%)	30.1	Pembrolizumab
**Park 2022**	South Korea	II	Esophageal squamous cell carcinoma	I and II	86	64 (39-76)	66 (42-83)	43 (96)	37 (90)	38.7	Durvalumab
**Kirkwood 2023**	International	III	Melanoma	IIB/C	790	62 (21–87)	61 (19–92)	322 (61.2)	161 (61.0)	15.8	Nivolumab
**Lian 2022**	China	II	Melanoma	I–III	145	57.7 (47.9–67.5)	57.4 (47.6–67.2)	30 (41.7)	22 (30.1)	26.3	Toripalimab

### Assessment of methodological bias

**Figure 2:**
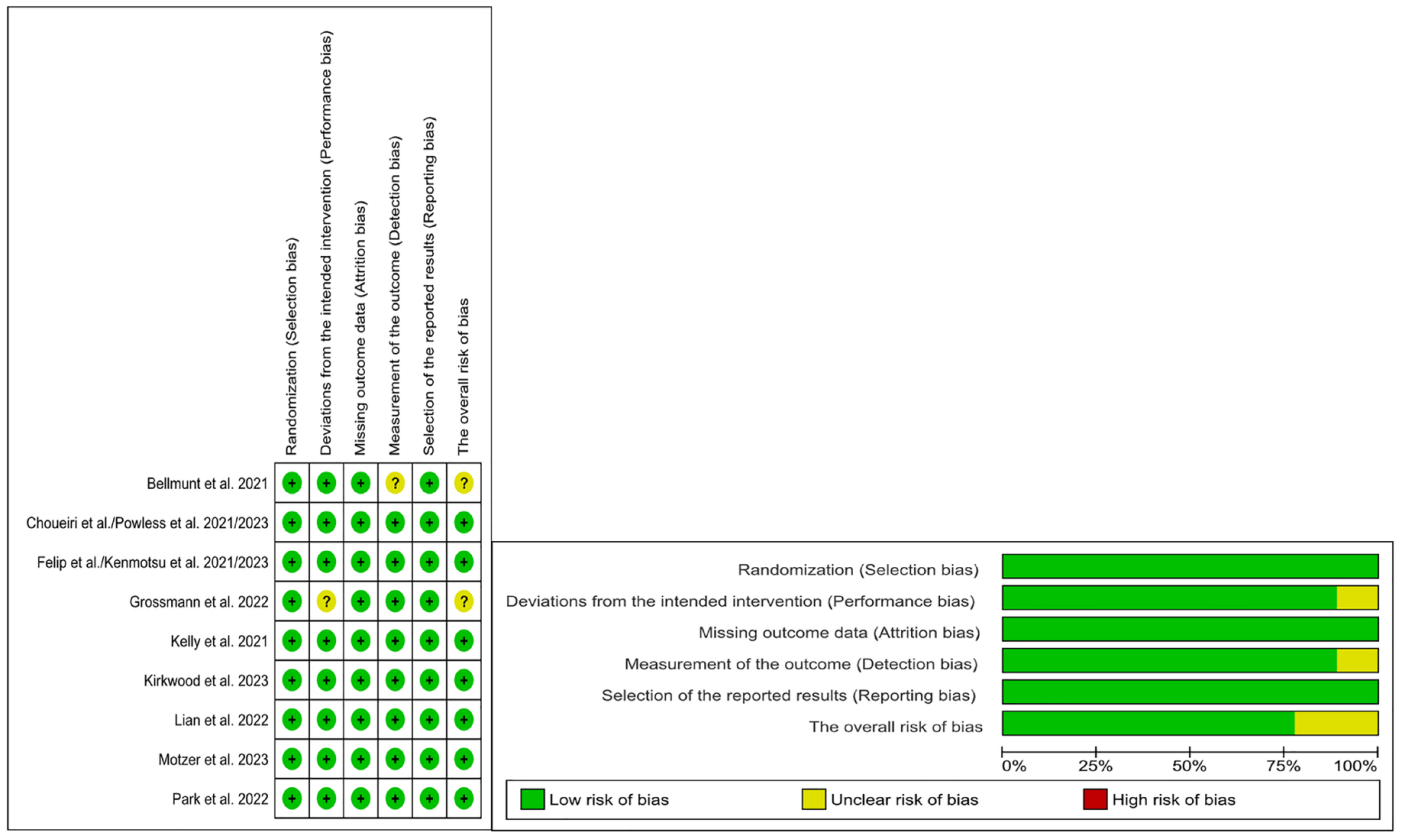
All included articles showed low risk of bias. There were two articles which showed some concern about potential bias. These two studies illustrated concerns in the following domains: detection bias and performance bias.

The selected articles were assessed using the Revised Cochrane Risk of Bias Assessment Tool 2 [21]. Two investigators independently evaluated each included study’s quality. Any discrepancies were settled through deliberations or by involving another investigator. A risk of bias summary is shown in Figure 2.

### Disease free survival (DFS)

**Figure 3:**
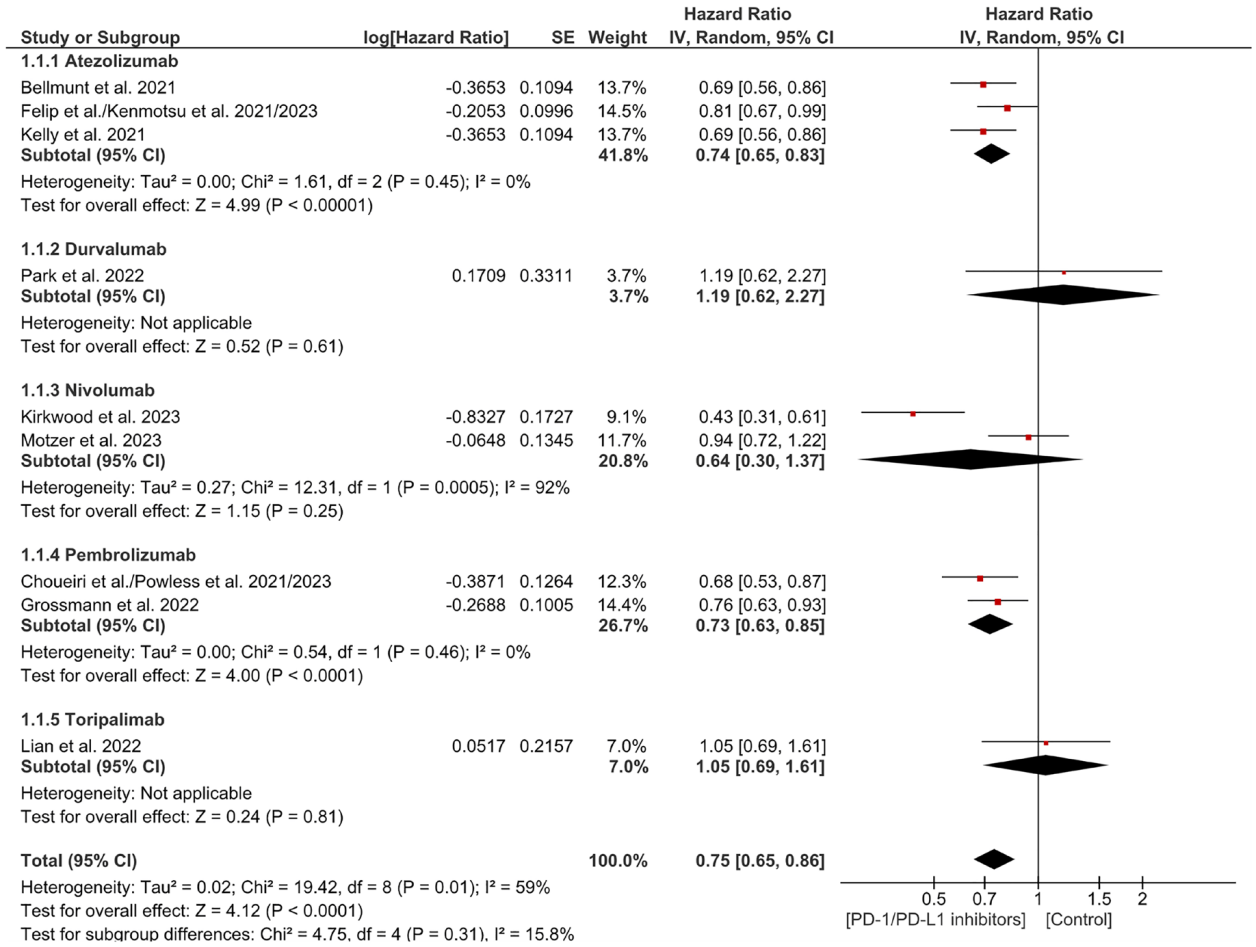
Forest plot for disease free survival.

**Figure 4:**
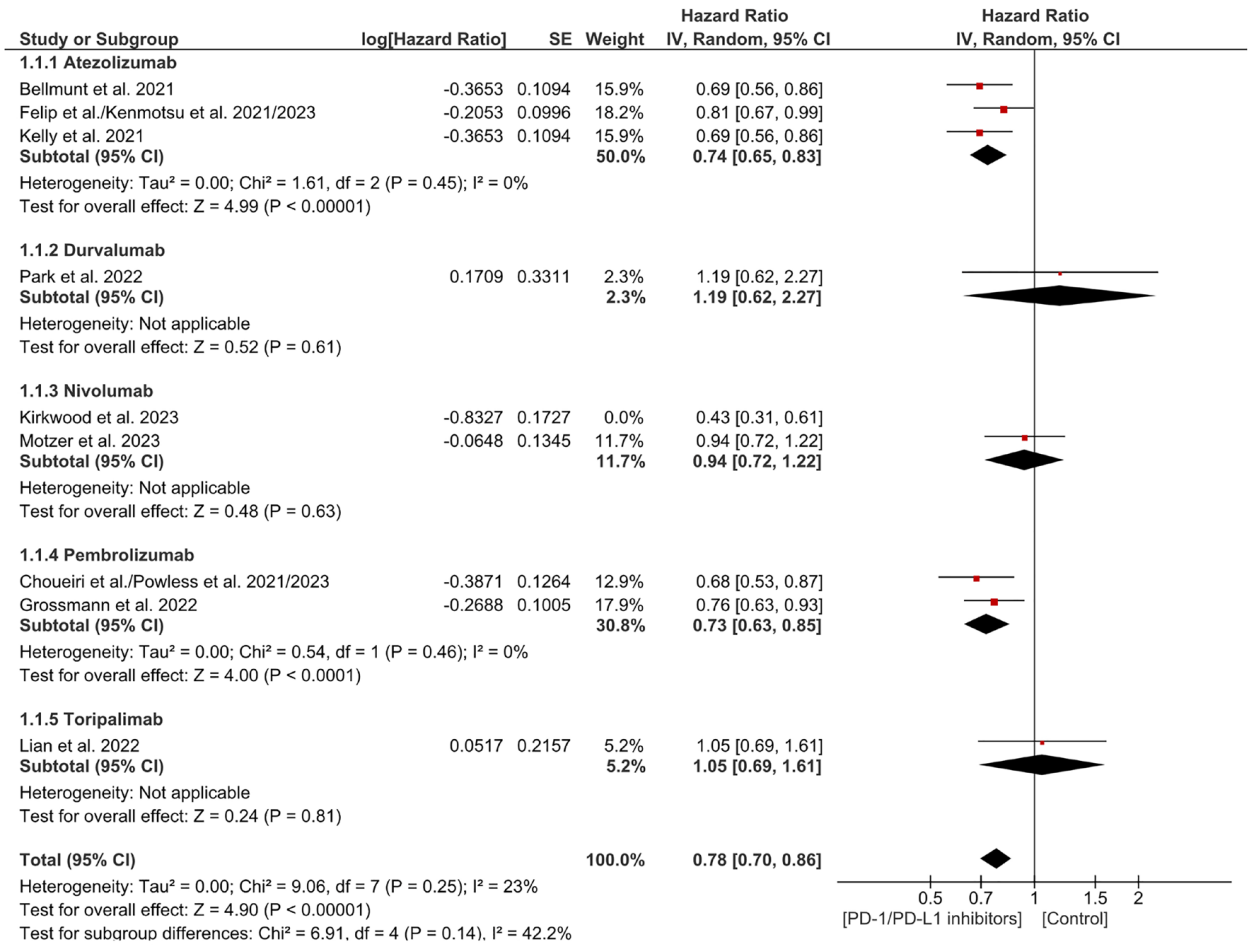
Sensitivity analysis for disease free survival.

**Figure 5:**
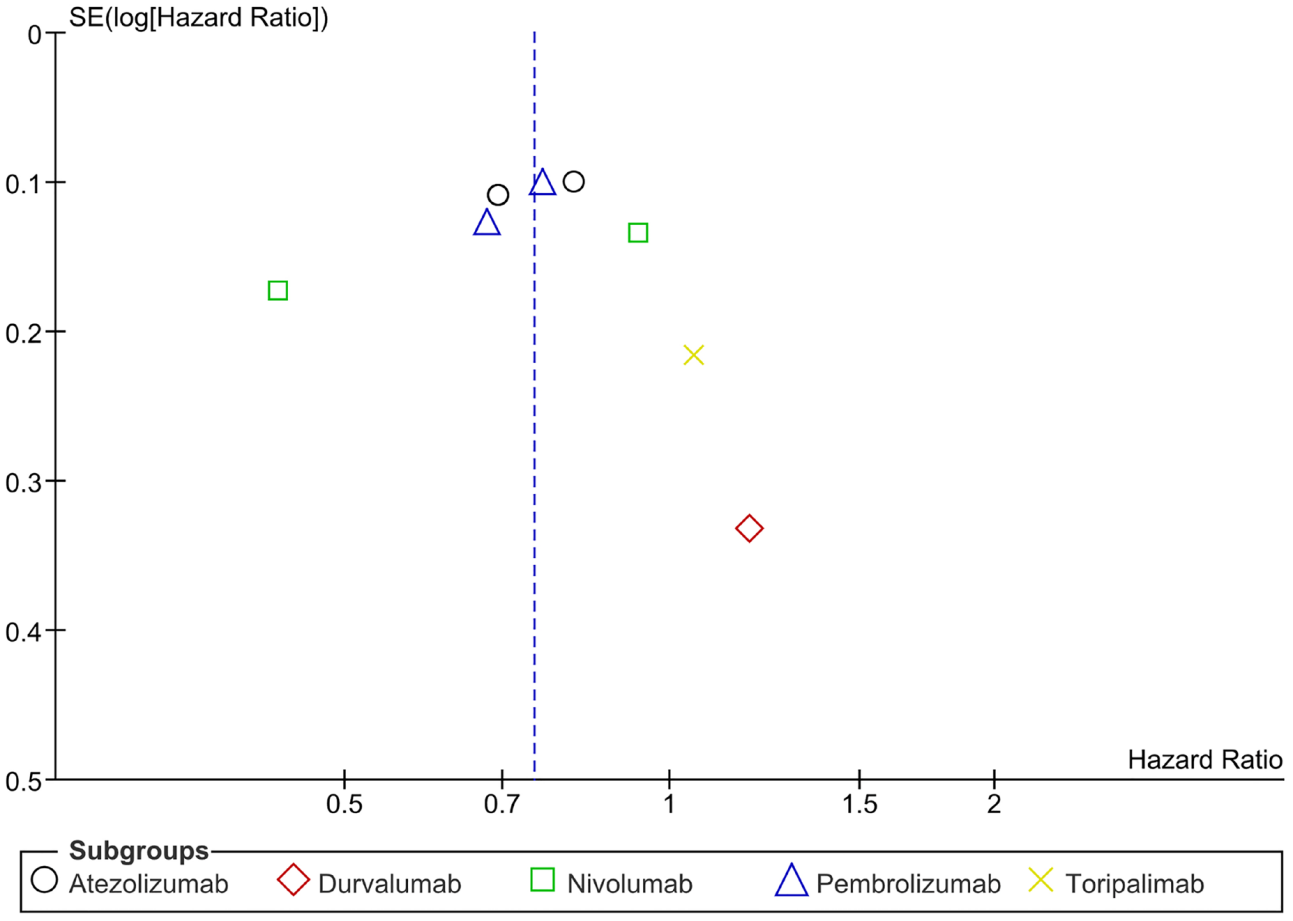
Funnel plot for disease free survival.

Disease-free survival is the time from treatment to recurrence or death. This analysis included 9 trials which showed a significant decrease in the likelihood of recurrence and death in the PD-1/PDL-1 arm in contrast to the control. As for the subgrouping, there was a substantial decrease in the risk of death and recurrence in Atezolizumab and pembrolizumab and no significant difference in the other subgroups. (HR = 0.75 CI 0.65–0.86; *P*-value less than 0.0001; I2 59%; *P*-value of Chi2 = 0.01) (Figure 3). There was significant heterogeneity. Sensitivity analysis was carried out which showed similar results with a decrease in the level of the heterogeneity. (HR = 0.78 CI 0.70–0.86; *P*-value less than 0.00001; I2 23%; *P*-value of Chi2 = 0.25) (Figure 4). No publication bias was noticed based on the funnel plot (Figure 5). Subgroup analyses by tumor type was limited by small study numbers and should be interpreted cautiously.

### Overall survival (OS)

**Figure 6:**
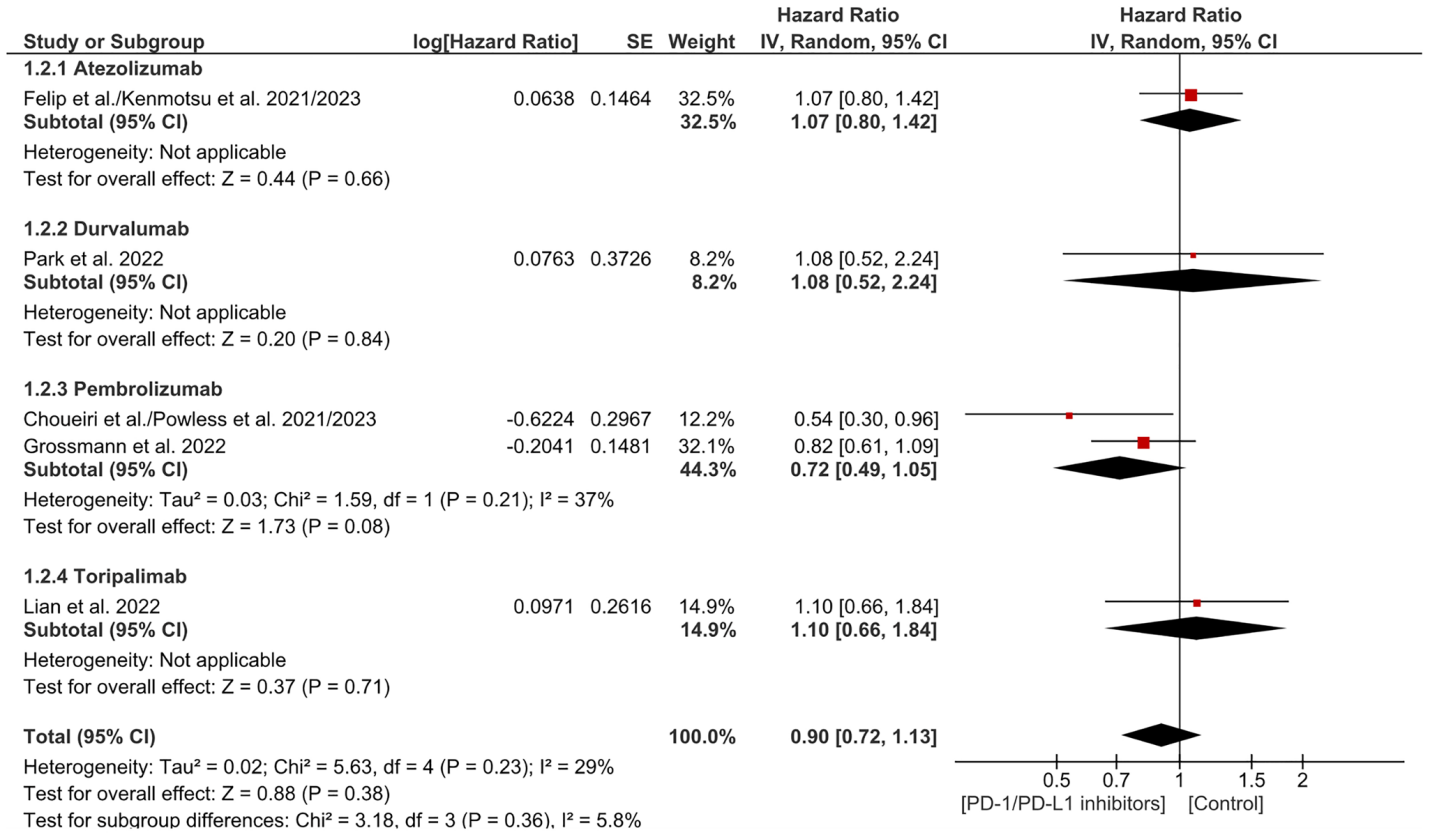
Forest plot for overall survival.

**Figure 7:**
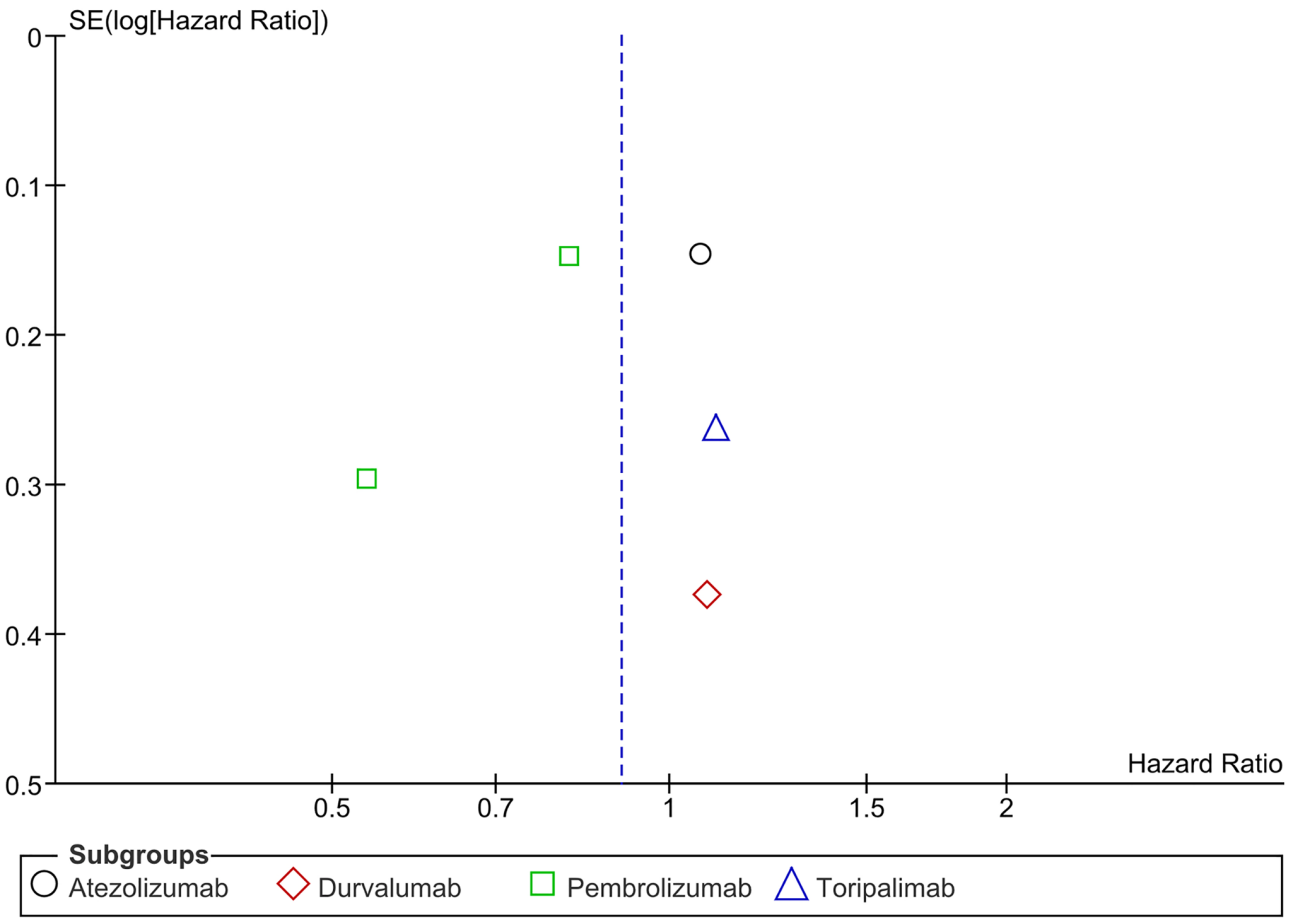
Funnel plot for overall survival.

Overall Survival (OS) is a method of describing prognosis of a certain disease. It is the time period since the patient has been diagnosed or received treatment to the time of death. Amongst the 13 included articles, a hazard ratio for OS was directly stated by 5 articles. No significant difference in OS was found between the two groups and no significant discrepancy in subgrouping (HR = 0.90, CI 0.72–1.13; *P*-value = 0.38; I2 29%; *P*-value of Chi2 = 0.23) (Figure 6). No significant heterogeneity was observed. The funnel plot was symmetrical (Figure 7).

### Distant metastasis free survival (DMFS)

**Figure 8:**
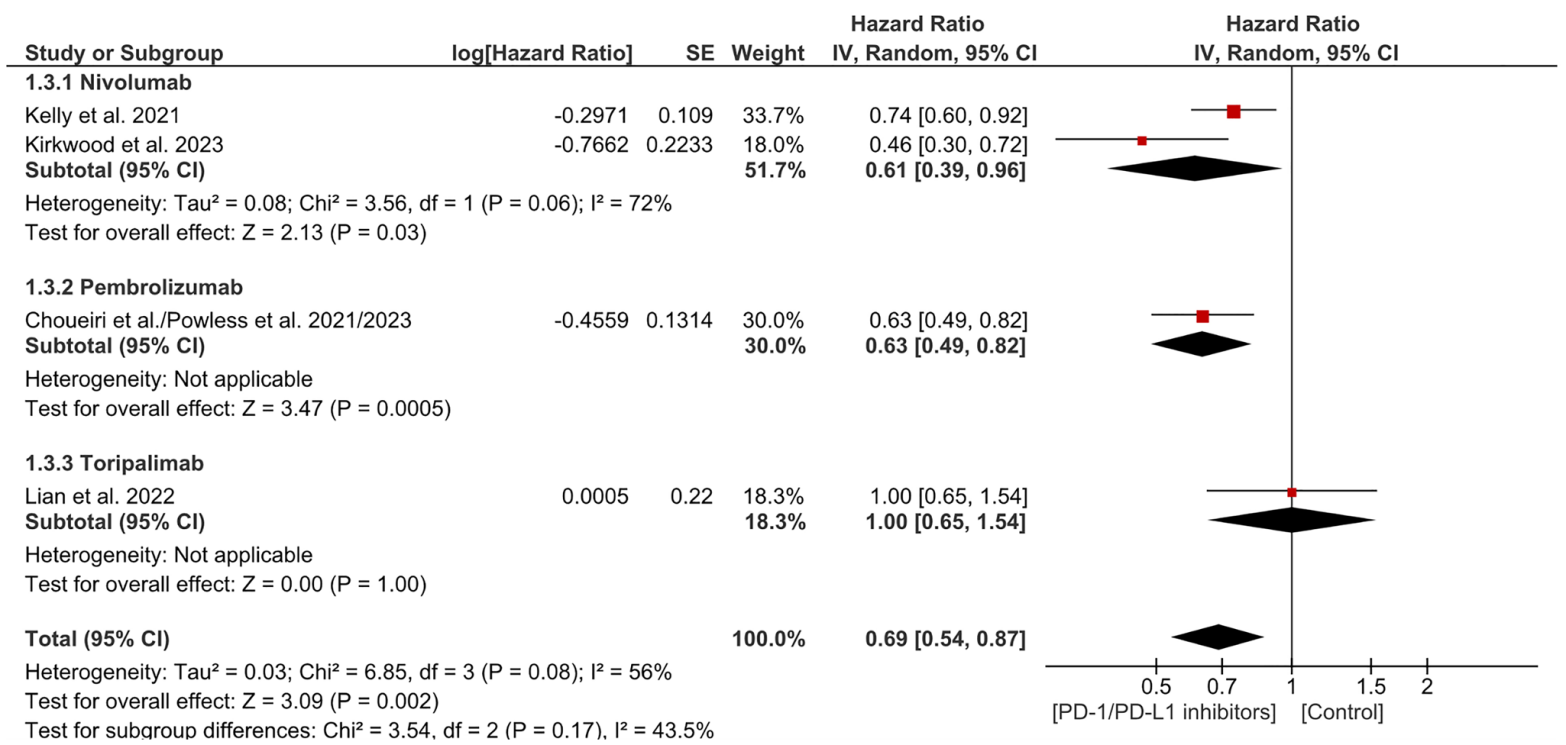
Forest plot for distant metastasis free survival.

**Figure 9:**
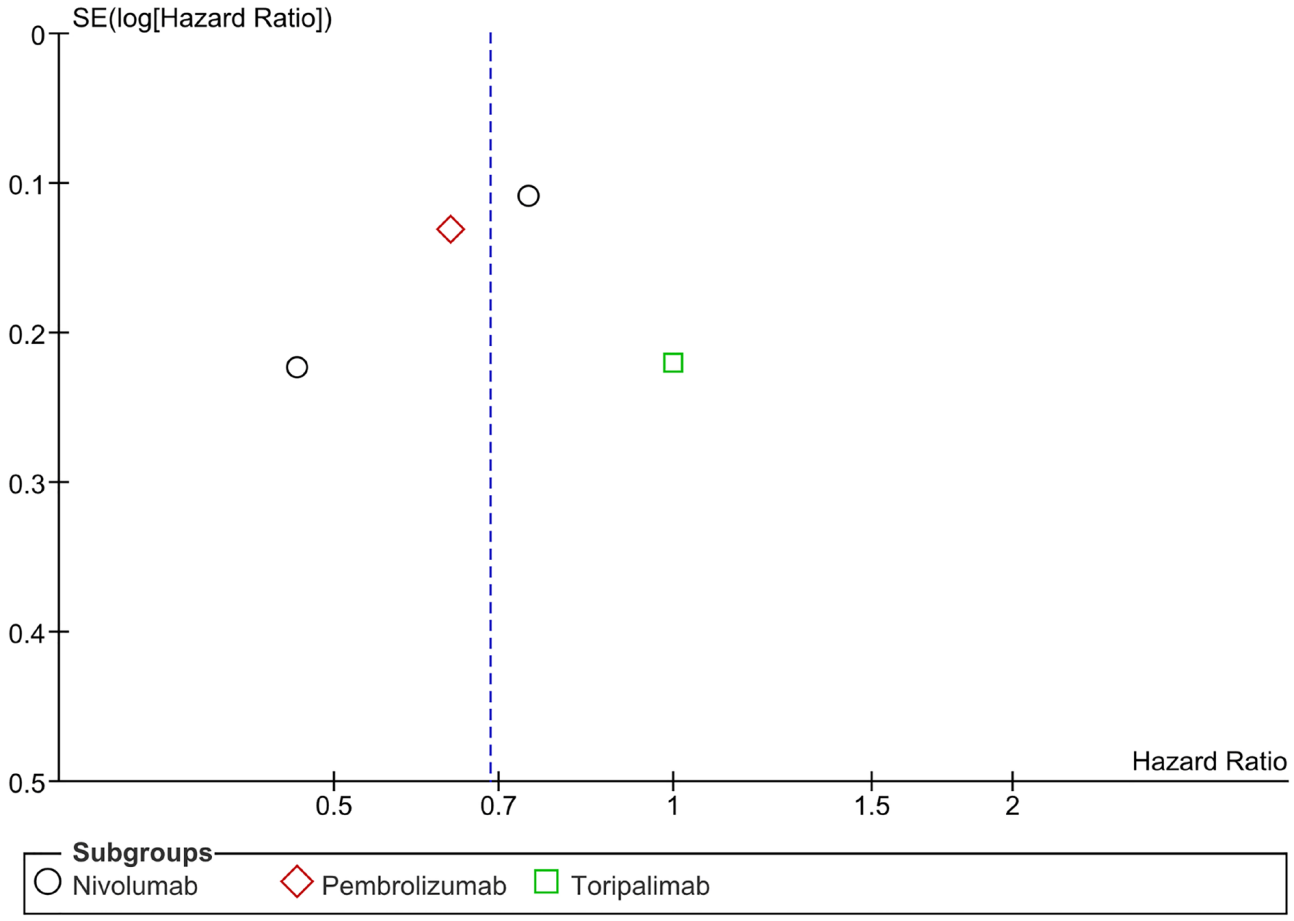
Funnel plot for distant metastasis free survival.

Distant Metastasis Free Survival (DMFS) is described as the time interval between the day of diagnosis of a cancer in a patient and the date on which distant metastases first appeared. In our study, PD-1/PDL-1 was found to significantly decrease the risk of distant metastasis reappearance or death due to any cause. In subgroup analysis, both Nivolumab and Pembrolizumab significantly reduced the risk of distant recurrence or death. There was no significant heterogeneity. (HR= 0.69 CI 0.54–0.87; *P*-value = 0.002; I2 56%; *P*-value of Chi2 = 0.08) (Figure 8). No publication bias was observed (Figure 9).

### Recurrence rate (RR)

**Figure 10:**
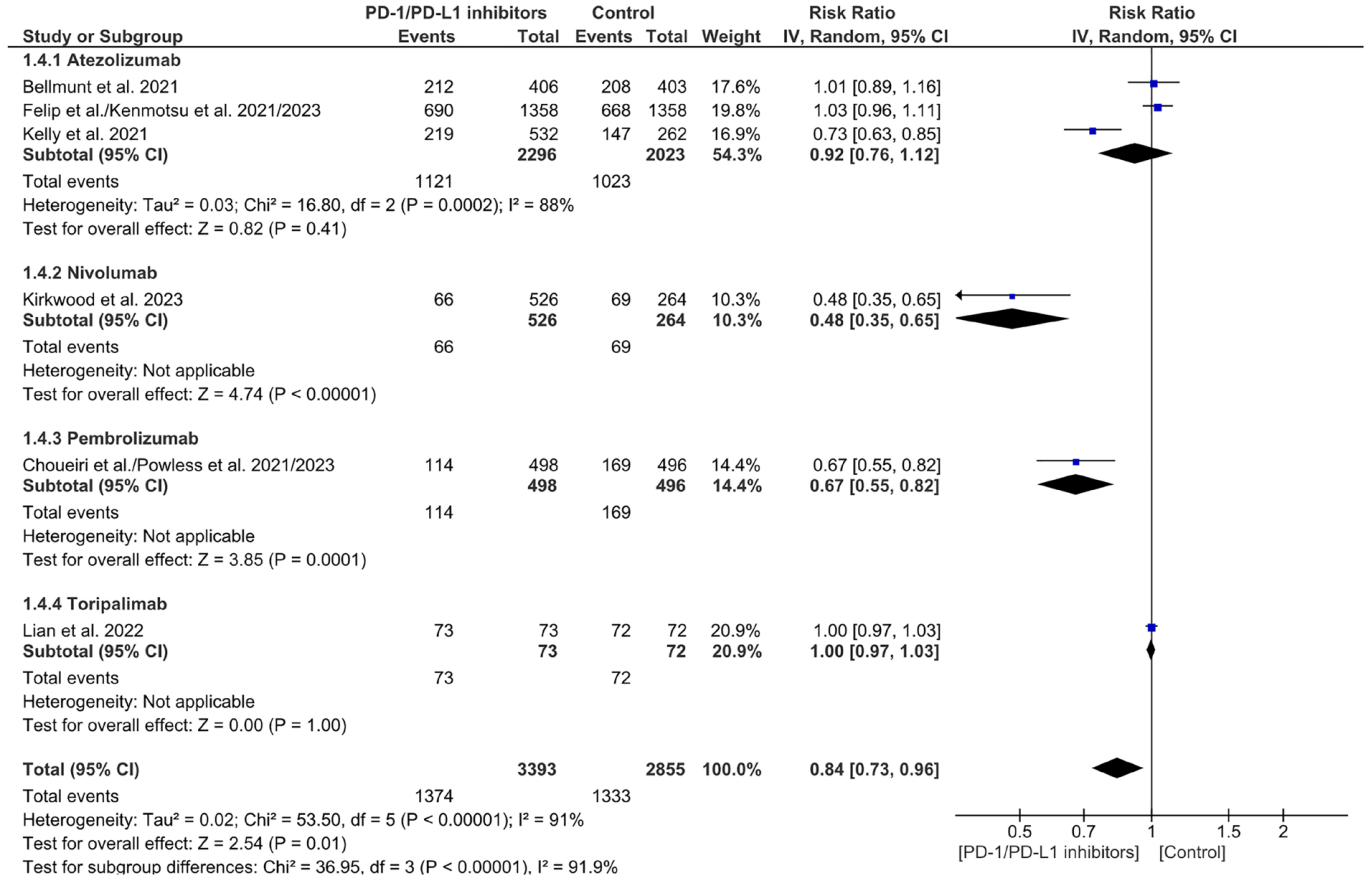
Forest plot for recurrence rate.

**Figure 11:**
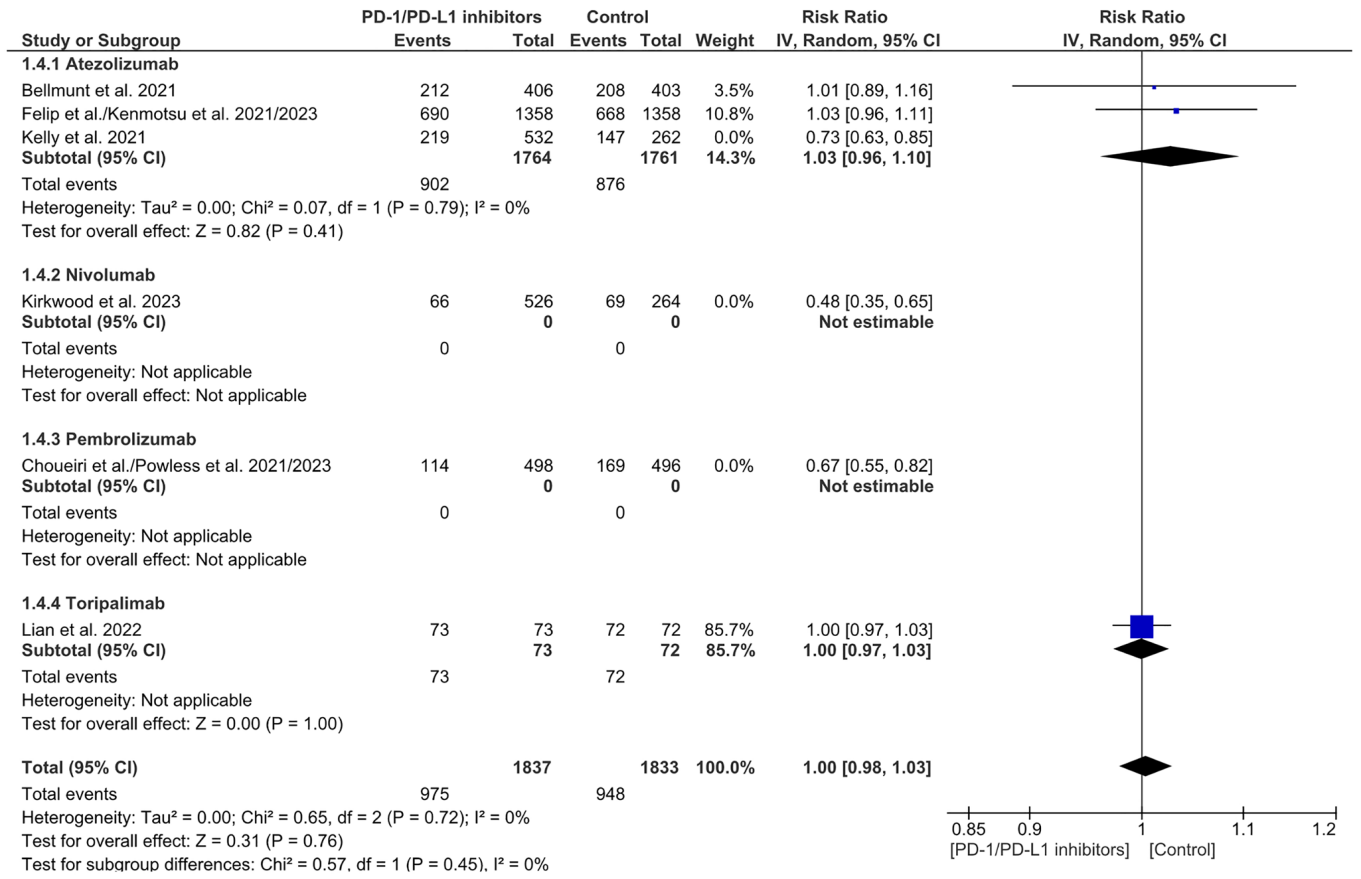
Sensitivity analysis for recurrence rate.

**Figure 12:**
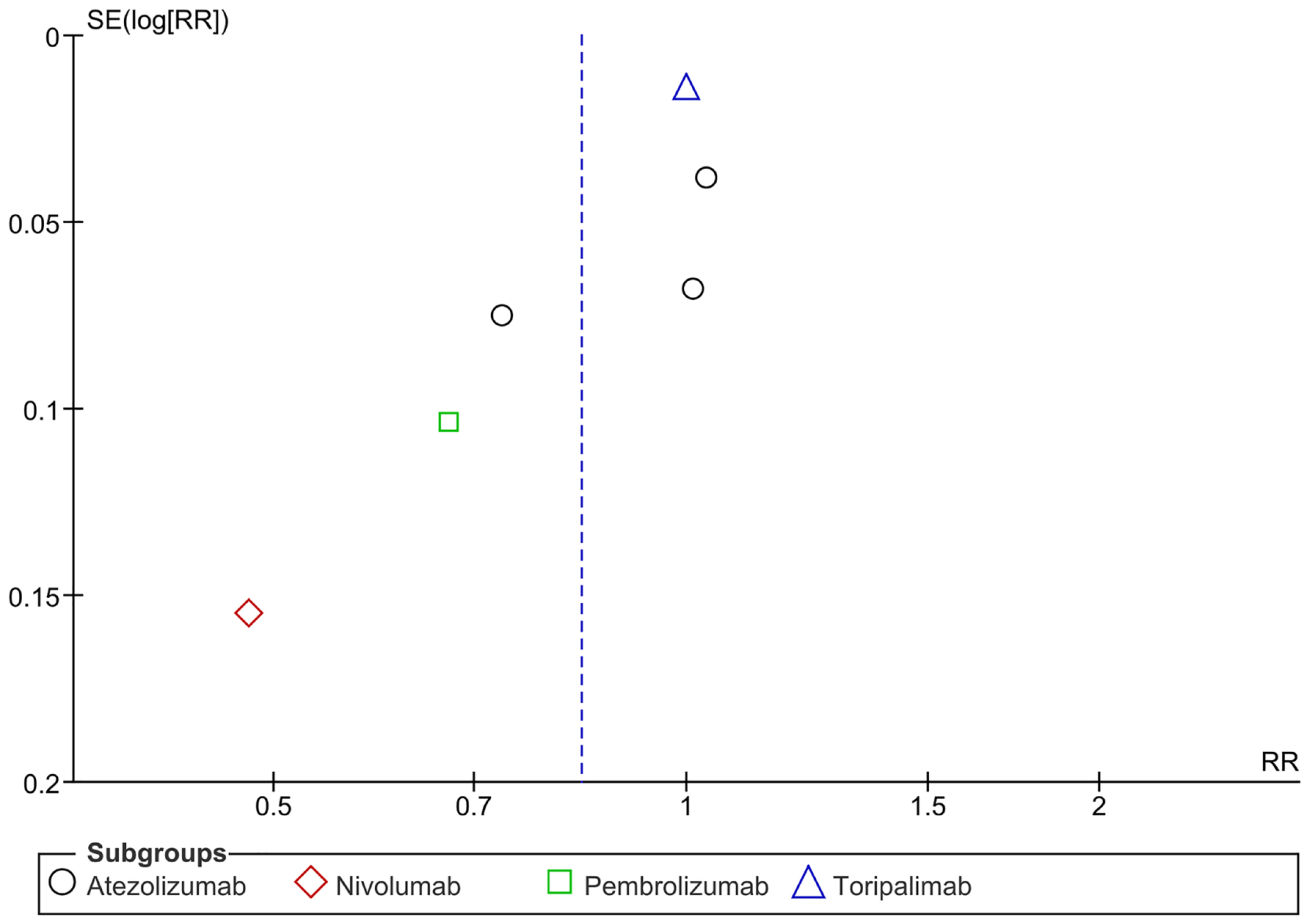
Funnel plot for recurrence rate.

Recurrence Rate (RR) is a measure of the recurrence of cancer. It was noticed that there’s a significant reduction in the recurrence rate in the overall PD-1/PDL-1 arm when compared to the control arm, with a similar result in the Nivolumab and Pembrolizumab subgroups. A significant heterogeneity and subgroup difference were observed in this analysis. (RR= 0.84 CI 0.73–0.96; *P*-value = 0.01; I2 91%; *P*-value of Chi2 less than 0.00001) (Figure 10). A sensitivity analysis revealed no discernible difference between PD-1/PDL-1 and the control with no notable distinction in the subgroup analysis. (RR = 1.00 CI 0.98-1.03; *P*-value = 0.76; I2 0%; *P*-value of Chi2 = 0.72) (Figure 11). No publication bias was observed (Figure 12).

### Incidence of adverse events (AEs)

**Figure 13:**
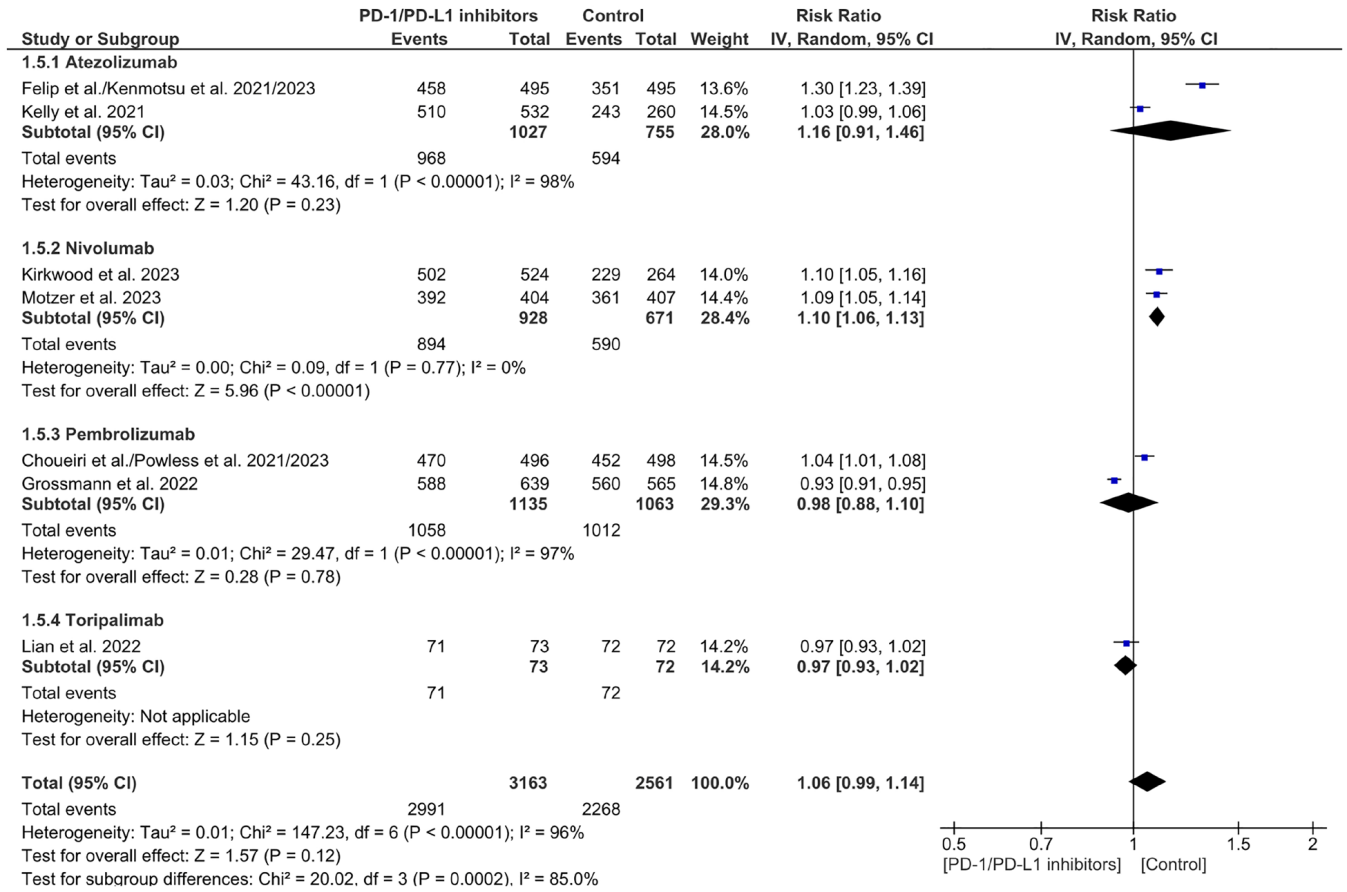
Forest plot for any adverse events.

A sum of 13 articles with 4,655 subjects included in the experimental category and 5,195 subjects in the PD-1/PD-L1 inhibitors arm were incorporated to analyze the occurrence of any adverse events and treatment-related adverse events. An analysis of the results showed that any adverse events were statistically significant in the investigational category in comparison to the control category (2991/3163 (94%) vs. 2268/2561 (88%), *P* < 0.00001) (Figure 13).

**Figure 14:**
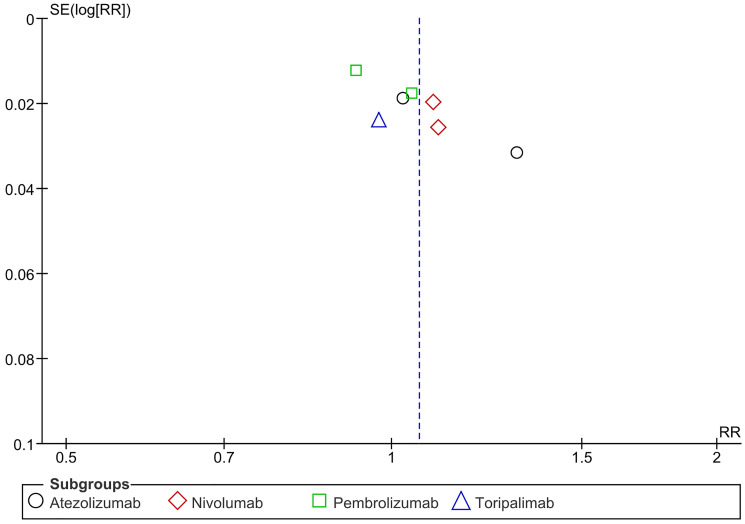
Funnel plot for any adverse events.

The most frequent adverse events reported were diarrhea (*n* = 8), fatigue (*n* = 8), pruritus (*n* = 8), hypothyroidism (*n* = 8), nausea (*n* = 8), rash (*n* = 8), and arthralgia (*n* = 7). There was a notable increase in any adverse events with Nivolumab. Overall and between groups, heterogeneity was significant in this analysis. (RR = 1.06 CI 0.99–1.14; *P*-value = 0.12; I2 96%; *P*-value of Chi2 less than 0.00001) (Figure 13). A symmetrical funnel plot; thus, no publication bias (Figure 14).

**Figure 15:**
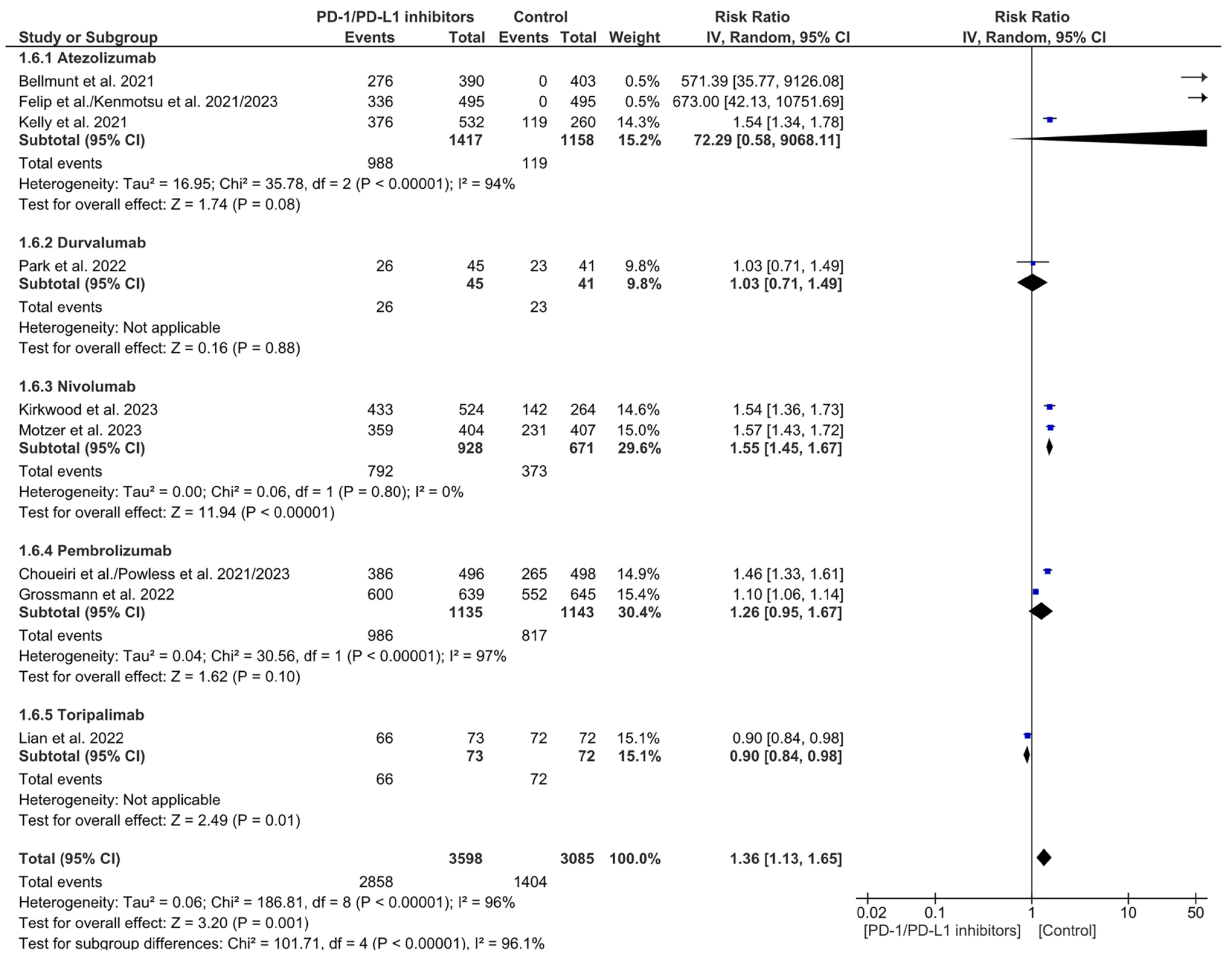
Forest plot for treatment-related adverse events.

**Figure 16:**
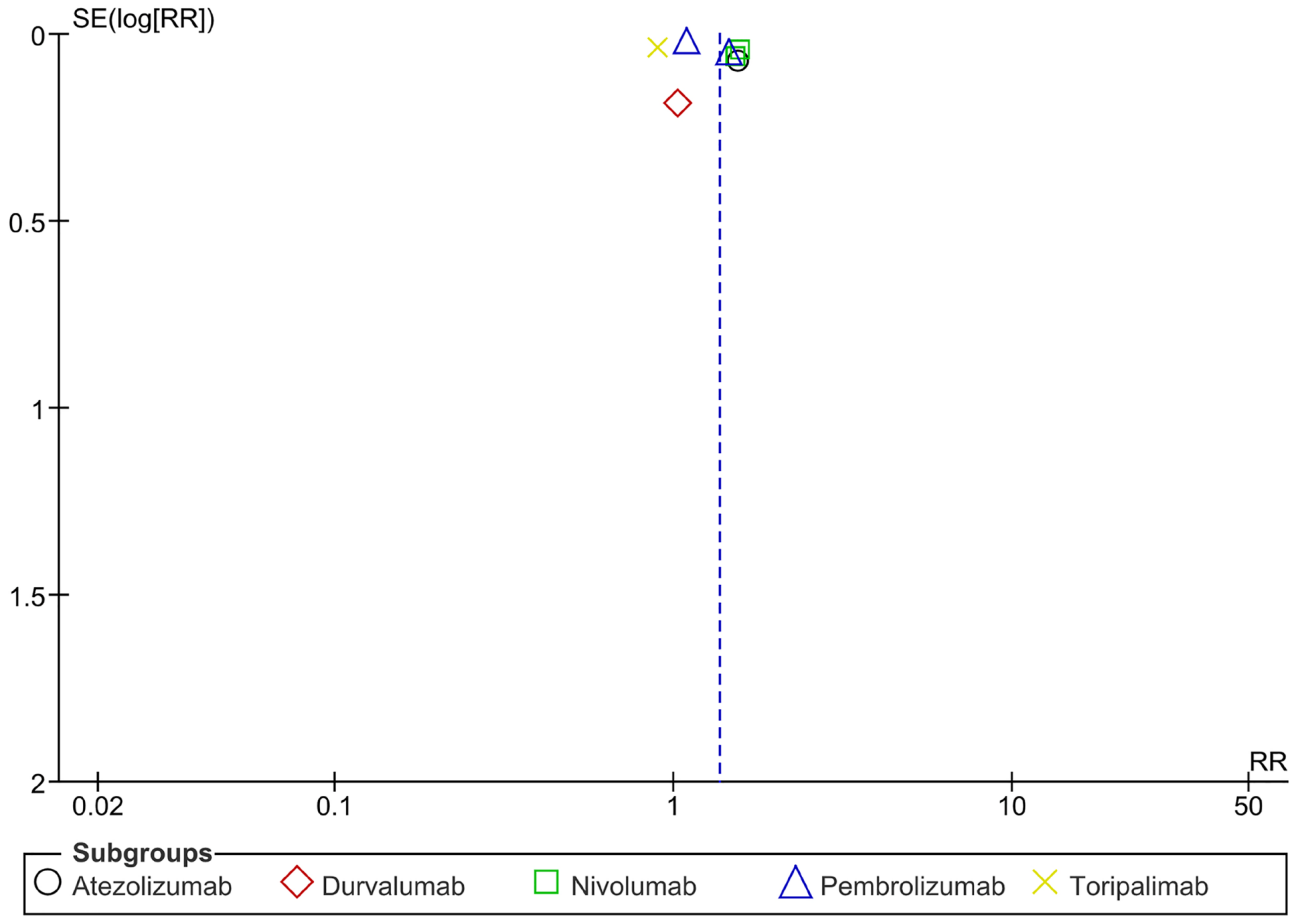
Funnel plot for treatment-related adverse events.

In relation to treatment-related adverse events, there was a notable rise in the rate of treatment-related adverse events in the PD-1/PDL-1 arm, especially with Nivolumab. However, there was a significant decrease in the treatment-related adverse events in Toripalimab. There was a significant overall heterogeneity and between subgroups. (RR = 1.36 CI 1.13–1.65; *P*-value = 0.001; I2 96%; *P*-value of Chi2 less than 0.00001) (Figure 15). No publication bias was detected (Figure 16).

### Publication bias

Visual analysis of the funnel plot was conducted to evaluate publication bias, and heterogeneity was measured using I2 and the *P*-value of Chi2.

## DISCUSSION

This meta-analysis synthesized data from 13 RCTs conducted between 2021 and 2023, encompassing 9,850 patients across multiple regions. The studies addressed the effectiveness of PD-1 and PD-L1 inhibitors as adjuvant treatments for solid tumors. The primary outcomes evaluated included disease-free survival (DFS), overall survival (OS), distant metastasis-free survival (DMFS), recurrence rates, and adverse events.

Worldwide, cancer is a leading cause of mortality, predominantly manifesting as solid tumors [1, 2]. Immune checkpoint inhibitors have become successful treatments because they improve the immune system’s capacity to recognize and combat cancer cells. These inhibitors, in particular, target the PD-1 and PD-L1 axis [7]. This study evaluated both the efficacy and safety of PD-1/PD-L1 inhibitors as adjuvant therapies for various solid tumors.

This analysis demonstrates that PD-1/PD-L1 inhibitors significantly improve disease-free survival (DFS) in patients with resected solid tumors. As it was demonstrated in the study on esophageal and gastroesophageal junction cancer, where DFS hazard ratio (HR) of 0.69 (95% CI 0.56–0.86), with a median DFS of 22.4 months in the treatment arm versus 11.0 months in the control [8]. Similarly, pembrolizumab showed a DFS HR of 0.76 (99.62% CI 0.59–0.99) in high-risk melanoma, supporting the role of these inhibitors in reducing recurrence. However, heterogeneity (I² = 59%) was noted, potentially due to variations in patient populations and cancer types [10]. Atezolizumab, for instance, did not significantly improve DFS in urothelial carcinoma (HR 0.93) [9], and nivolumab plus ipilimumab showed non-significant results in renal cell carcinoma (HR 0.94) [11]. Sensitivity analysis reduced heterogeneity to 23%, confirming the overall positive impact of these inhibitors. These findings suggest that adjuvant PD-1 and PD-L1 inhibitors may reduce recurrence risk in selected high-risk solid tumors. However, the magnitude of benefit appears to differ between tumor types, and the available evidence within each subgroup remains limited.

The findings indicate no significant difference in OS between treatment arms (HR = 0.90, CI 0.72–1.13, *p* = 0.38), with low heterogeneity across subgroups (I² = 29%). However, specific trials indicate trends favoring pembrolizumab in renal cell carcinoma. In one study, fewer deaths occurred in the pembrolizumab group (18 vs. 33 in placebo), with a hazard ratio for death of 0.54 (95% CI 0.30–0.96), and the 24-month OS estimate was higher (96.6% vs. 93.5%) [15]. The KEYNOTE-564 trial further supported pembrolizumab’s survival benefit (HR 0.52, 95% CI 0.31–0.86), but statistical significance was not reached, and longer follow-up is required [17]. Conversely, durvalumab in esophageal squamous cell carcinoma did not show significant OS improvement (HR 1.08, 95% CI 0.52–2.24, *p* = 0.85) [18], and toripalimab in mucosal melanoma did not demonstrate a significant OS benefit (HR 1.11, 95% CI 0.66–1.84) [20]. Pembrolizumab shows promise in renal cell carcinoma, but further follow-up is essential. The need for a longer period of follow-up, plus the fact that some trials were not essentially powered to observe OS or examined OS as a secondary endpoint, might have resulted in the lack of benefit, as seen with durvalumab and toripalimab. Moreover, it is necessary to refine patient selection and investigate biomarkers to predict response. Research in the future should focus on long-term outcomes and combination therapies to enhance survival.

PD-1/PD-L1 inhibitors significantly reduce the risk of distant metastasis or death (HR = 0.69, CI 0.54–0.87, *p* = 0.002). Both nivolumab and pembrolizumab showed significant benefits in lowering distant recurrence with no publication bias. In esophageal cancer, adjuvant nivolumab improved distant metastasis-free survival (DMFS) to 28.3 months vs. 17.6 months for placebo (HR 0.74, 95% CI 0.60–0.92) [8]. Similarly, in renal cell carcinoma, pembrolizumab showed better DMFS at 30 months (HR 0.63, 95% CI 0.49–0.82) [17]. In resected melanoma, nivolumab reduced distant recurrence (HR 0.47, 95% CI 0.30–0.72) [19]. However, toripalimab in mucosal melanoma did not improve DMFS compared to high-dose interferon-α2b (HR 1.00, 95% CI 0.65–1.54) [20]. Nivolumab and pembrolizumab show clear benefits in reducing distant metastasis in esophageal, renal, and melanoma cancers.

According to this review, the intervention group experienced a statistically significant increase in any adverse events when compared to the control group (94% vs. 88%, *p* < 0.00001). This suggests that adverse events were more common in the treatment group’s patients, requiring proper monitoring and care. In accordance with established PD-1/PD-L1 inhibitor side effect profiles, rash, nausea, arthralgia, lethargy, diarrhea, pruritus, and hypothyroidism are risks to develop. Adverse events were more common in the Nivolumab group as compared to other groups. Conversely, adverse events associated with Toripalimab had a lower incidence, indicating differences in safety pro-files among the drugs. Significant heterogeneity was observed (I² = 96%; *p* < 0.00001 for Chi²), caused by some variations in patient populations, study design, and treatment regimens. Despite these variations, symmetrical funnel plots suggest no publication bias, fortifying the quality of the findings. Nivolumab was investigated in three studies among esophageal or gastroesophageal junction cancer, Renal cell carcinoma, and Melanoma. In esophageal and gastroesophageal cancer, Kelly et al reported a 34% incidence rate of grade 3 or 4 adverse events of any cause and a 30% rate of serious adverse events of any grade. In renal cell carcinoma, the study by Motzer et al used nivolumab plus ipilimumab and reported a 38% grade 3–5 adverse events and 32% all-cause adverse events. Moreover, Kirkwood et al reported an 82.6% incidence rate of Any-grade treatment-related adverse events with nivolumab in melanoma treatment and a 10.3% rate of grade 3 or 4 events [8, 11, 19].

This review revealed increased adverse events associated with PD-1/PD-L1, especially nivolumab, that are statistically significant. Despite that, most common adverse events were similar to the known side effect profiles, the increased risk requires careful observation and management of patients receiving those drugs. The long-term adverse events and their impact on patient’s quality of life require further investigations.

This systematic review and meta-analysis have several limitations. First, interpretation of cancer-specific outcomes is constrained by the small number of randomized trials available for each tumor type and treatment regimen; therefore, subgroup analyses should be considered exploratory rather than confirmatory. Second, most included studies had relatively short follow-up durations, which may account for the absence of a clear overall survival benefit.

In addition, although funnel plot analyses did not indicate publication bias for most outcomes, unpublished studies with null or negative results cannot be excluded, potentially leading to an overestimation of treatment effects. Heterogeneity in treatment protocols across trials further limits the ability to identify optimal regimens for specific cancer types, and evidence remains limited for broader solid tumor populations. Addressing these limitations in future studies is essential to strengthen the evidence base and guide the appropriate use of PD-1/PD-L1 inhibitors as adjuvant therapies in solid cancers.

## MATERIALS AND METHODS

This review followed PRISMA guidelines [22], and was prospectively registered in PROSPERO (CRD42024563699). PubMed, Web of Science, and Google Scholar were searched in two stages using predefined Medical Subject Headings and keywords related to PD-1/PD-L1 inhibitors and solid cancers. Other studies were obtained by conducting reference list checks on the included studies as well as other published systematic reviews and meta-analyses [23, 24].

### Inclusion and exclusion criteria

Eligible studies enrolled patients with early-stage solid tumors receiving adjuvant PD-1 or PD-L1 inhibitors and reported survival or safety outcomes. Only randomized controlled trials published in English full-text format were included. The review excluded studies involving liquid cancers, and non-randomized studies such as editorials, letters, commentaries, and systematic reviews. Additionally, studies that did not report the specified outcomes, those published in languages other than English, single-arm studies, and Phase I trials were also excluded. This approach ensured the review focused on robust and comparable data.

### Selection of articles and data extraction

Two pairs of reviewers independently screened titles and abstracts, assessed full texts, and extracted data. Disagreements were resolved by discussion. The extracted data include study characteristics (publication year, author, country, study phase, sample size, cancer type, and stage), patient demographics (age, sex), intervention details (type and dosage of PD-1/PD-L1 inhibitors), and Several outcomes (disease-free survival, overall survival, distant metastasis free survival, recurrence rate, Adverse Events).

### Data analysis

The meta-analysis and forest plot generation were carried out using Review Manager Software version 5.4. All findings were examined utilizing a random-effects model. Risk ratios (RR) were reported for recurrence rate, any adverse event, and treatment-related adverse events. The hazard ratio was used to assess disease-free survival, overall survival, and distant metastasis-free survival. Any trial with an outcome that is defined as the time from randomization to the earliest date of disease recurrence or death, whichever occurred first, was considered disease-free survival, and distant-free survival was defined as the date of first distant recurrence or death from randomization. Overall survival was defined as any death occurring after the period of randomization. Visual inspection of the funnel plot was utilized to evaluate publication bias and heterogeneity was estimated using the I2 and the Chi2 *P*-value. Statistical significance was determined using a *P*-value less than 0.05 and a 95% confidence interval. Whenever possible, a sensitivity analysis was done when significant heterogeneity was observed.

## CONCLUSIONS

This systematic review and meta-analysis of 13 randomized controlled trials including 9,850 patients shows that adjuvant PD-1 and PD-L1 inhibitors improve disease-free and distant metastasis-free survival in patients with high-risk solid tumors. No clear overall survival advantage has yet been demonstrated, likely due to limited follow-up. These agents are associated with higher rates of treatment-related adverse events, which require careful monitoring. Because the number of studies within each cancer type was small, cancer-specific conclusions should be interpreted with caution. Larger trials with longer follow-up are needed to define which patient groups derive the greatest benefit.

## Abbreviations

ICB: Immune checkpoint blockade
OS: Overall survival
ICIs: Immune checkpoint inhibitors
PD-L1: Programmed cell death ligand-1
PD-1: Programmed cell death protein-1
PRISMA: Preferred Reporting Items for Systematic Reviews and Meta-Analyses
PROSPERO: International Prospective Register of Systematic Reviews
MeSH: Medical Subject Headings
DFS: Disease-Free Survival
ORR: Overall Response Rate
PFS: Progression-Free Survival
OS: Overall Survival
RR: Recurrence Rate/Risk Ratio
AEs: Adverse Event
Chi²: Chi-Square Test
CI: Confidence Interval
DMFS: Distant Metastasis-Free Survival
HR: Hazard Ratio
I²: Heterogeneity Index
RCT: Randomized Controlled Trial
